# Pharmacotherapies to tics: a systematic review

**DOI:** 10.18632/oncotarget.25080

**Published:** 2018-06-15

**Authors:** Zuojie Zhang, Chunsong Yang, Ling-Li Zhang, Qiusha Yi, Bo Liu, Jing Zeng, Dan Yu

**Affiliations:** ^1^ Department of Pharmacy, Evidence-Based Pharmacy Center, West China Second Hospital, Sichuan University, Chengdu, China; ^2^ Key Laboratory of Birth Defects and Related Diseases of Women and Children (Sichuan University), Ministry of Education, Chengdu, China; ^3^ West China School of Pharmacy, Sichuan University, Chengdu, China; ^4^ Department of Neurology, West China Second Hospital, Sichuan University, Chengdu, China

**Keywords:** tics, efficacy, meta-analysis, review, pharmacotherapy

## Abstract

The efficacy of all pharmacotherapies for patients suffering from tics were unclear. Literatures were searched from Medline, Embase, The Cochrane Library, and four Chinese databases. The primary efficacy outcome scale was defined as the Yale Global Tic Severity Scale (YGTSS). Overall estimates of pooled weighted mean difference (WMD) with 95% confidence interval (CI) were calculated for each outcome measure. A total of 53 trials were included. Meta-analysis suggested that alpha-2 adrenergic agonist agents and atypical antipsychotic agents were effective in improving tics, which included the maximum number of trials. Typical antipsychotic agents were associated with severer side-effects than alpha-2 adrenergic agonist agents. Besides, Traditional Chinese Medicine showed positive effects in YGTSS (NingDong Granule: WMD=-7.100, 95% CI, -10.430- -3.770; 5-Ling Granule: WMD=-11.300, 95% CI, -14.208- -8.392), while glutamate modulators (D-serine, N-Acetylcysteine and riluzole) might not be working. In summary, alpha-2 adrenergic agonist agents were associated with the optimal weigh between efficacy and safety. However, the significant factor of limited trials and sample sizes discounted these findings. Further better studies are necessary to ascertain them.

## INTRODUCTION

Tourette's syndrome (TS) and chronic tic disorder (CTD) are neurodevelopmental diseases characterised by the appearance of at least one time phonic/vocal and motor tics within one year [1, 2]. The onset age is typically 5 to 6 years old and the worst age is 10 to 12 years old [3]. Two epidemiological studies discovered that TS was diagnosed in around 0.3% to 1% of school age children [4, 5].

Tics are frequently comorbid with considerable diseases such as attention deficit hyperactivity disorder (ADHD), autism spectrum disorder, anxiety, depression, and obsessive compulsive disorder [6–10]. The nature damage of tics and corresponding comorbidities have significant adverse implications on quality of life and social environment [11–13]. In addition, caregivers undergo enormous burden and possibly tend to develop psychological diseases [14]. Children as well as caregivers emphasized the importance of tics reduction. So, seeking for appropriate treatments to improve tics in time is a clinical priority.

There are several types of interventions for tics including pharmacotherapies, behavioural therapies, and physical therapies, but the most widely used and mainstay treatment remains pharmacotherapy [15, 16]. And among pharmacotherapies, antipsychotics were generally recognized. Although, several cognitive behavioural therapies, such as cognitive-behavioral approach, cognitive psychophysiological treatment, behavioural therapy, and habit reversal therapy, were supported to use in tics with many high-quality evidences and some of them were inserted in the Canadian guidelines for evidence-based practices [17, 18, 19, 20], surveys found that these approaches might be poor to access and difficult to use [21]. In addition, clinical trials of these treatments were impeded by several reasons including failing to blind participants and the reasonability of their use [19].

As to western medicine, none were developed to target at tics, although tics have been considered as biological conditions [22]. Despite this, these treatments have been widely applied to suppress tics, including alpha-2 adrenergic agonist agent, antipsychotic drugs, anticonvulsant, analgesic, glutamate agonist, etc. Also, many clinical trials and systemic reviews were conducted to evaluate the efficacy of pharmacotherapies for tics [23–25]. However, these studies reached mixed results. For several interventions, some evidences suggested that they were effective in decreasing disease burden, while others yielded the opposite results [26–30]. And none has summarized all pharmacotherapies. Currently, more and more powerful national groups, such as the American Academy of Child and Adolescent Psychiatry, the Canadian Academy of Child and Adolescent Psychiatry, the European Society for the Study of Tourette Syndrome, and the Chinese Medical Association, established clinical guidelines to introduce the use of pharmacotherapy for tics [21, 23]. These recommendations demonstrated the increasing focus on the use of pharmacotherapies for tics, but many first-line treatments were associated with few evidences.

Therefore, in order to improve previous research, all trials were collected to evaluate the efficacy and safety of pharmacotherapies for tics patients, in which pharmacotherapies were compared with placebo or any other pharmacotherapies.

## RESULTS

### Study characteristics

**Figure 1:**
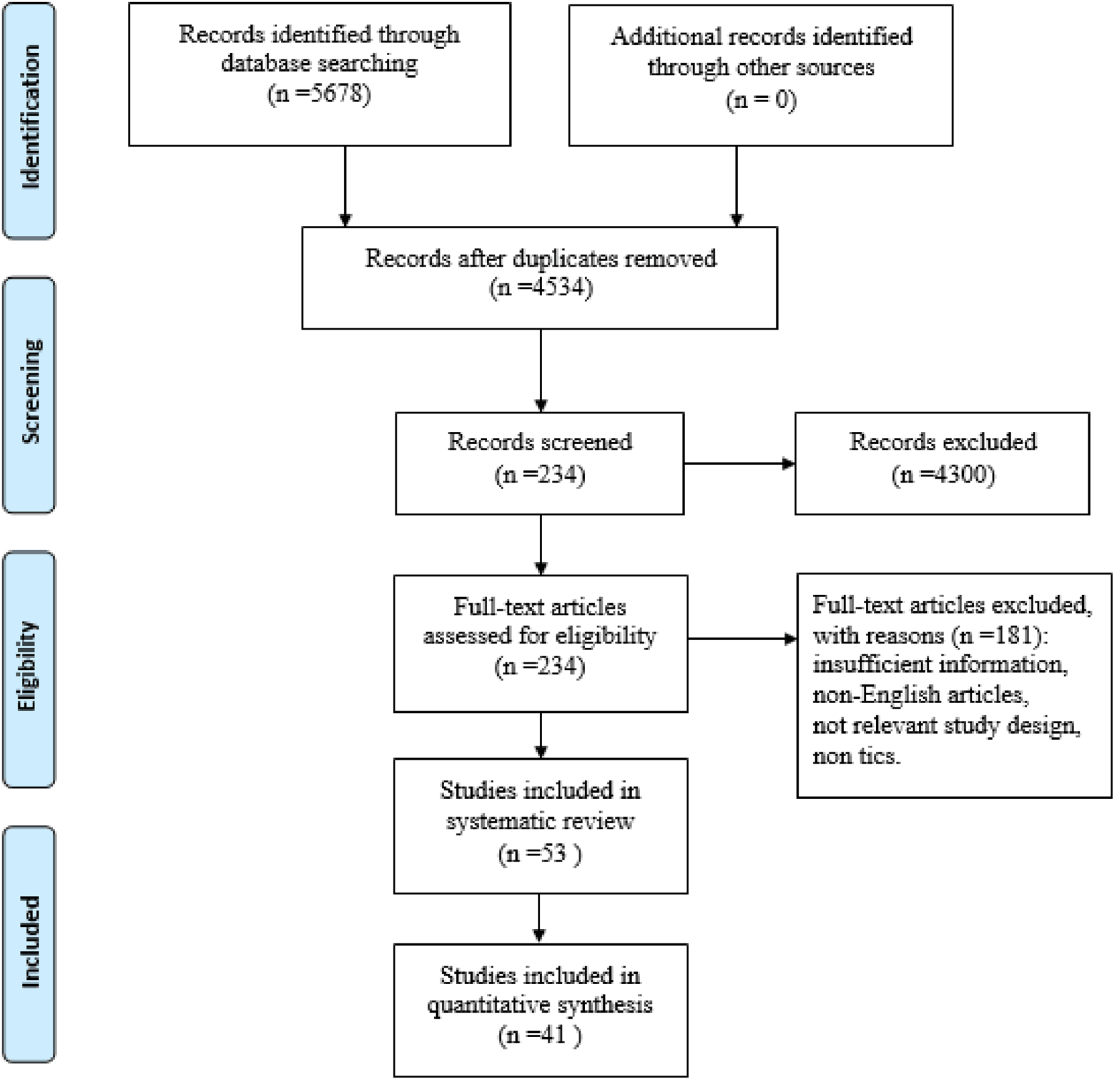
Flow chart of meta-analysis

Fifty-three studies [26–29, 31–79] including 3155 patients were identified after literature selection, of which 15 studies were associated with mixed participants (children and adult). Types of treatments and numbers of included research were summarized in Table 1. PRISMA flowchart was displayed in Figure 1.

**Table 1:** Types of treatments and numbers of researches included

Intervention	Number of studies
Alpha-2 adrenergic agonist agent	
Guanfacine	2
Clonidine	7
Clonidine Patch	1
Lofexidine	1
Analgesic	
Naltrexone	1
Propoxyphene	1
Anticonvulsant	
Levetiracetam	2
Topiramate	1
Antidepressant	
Fluoxetine	1
Fluvoxamine	1
Desipramine	2
Deprenyl	1
Antipsychotic agent	
Typical Neuroleptics	
Haloperidol plus Trihexyphenidyl	2
Haloperidol	3
Pimozide	5
Sulpiride	1
Atypical Neuroleptics	
Ziprasidone	1
Risperidone	5
Aripiprazole	2
Tiapride	2
Olanzapine	1
Cannabis	
Delta-9-tetrahydrocannabinol	1
CNS stimulant	
Methylphenidate	5
Dextroamphetamine	1
Methylphenidate plus Clonidine	1
Cholinoceptor blocking drugs	
Mecamylamine	1
Dopaminergic agent	
Pergolide	2
Pramipexole	1
Talipexole	1
Gamma-aminobutyric acidB receptor agonist	
Baclofen	1
Glutamate agonist and antagonist	
D-serine	1
N-Acetylcysteine	1
Riluzole	1
Selective norepinephrine reuptake inhibitor	
Atomoxetine	2
Smoking cessation agent	
Nicotine patch	2
Smoking cessation agent plus Antipsychotic drugs	
Nicotine patch plus Haloperidol	1
Traditional Chinese medicine	
NingDong Granule	1
5-Ling Granule	1
Ningdong Granule plus Haloperidol	1
Qufeng Zhidong Recipe	2
5HT3-receptor antagonists	
Ondansetron	1
Metoclopramide	1

The characteristics of included studies were depicted in Table 2. Outcome data could not be available from 12 trials even by contacting relevant authors, so they were excluded from meta-analyses. Specific reasons were displayed as follows:1) 5 studies did not reported any of the four outcomes included in this meta-analysis [31, 34, 43, 53, 65]; 2) 1 study only reported whether the differences between experimental group and control group were significant, but it lacked outcome data [36]; 3) 2 studies only reported outcome data at endpoint without scores at baseline [33, 35]; 4) 4 studies only reported mean improvement data but weighted mean differences (WMD) with the 95% confidence interval (CI) were failed to be received [42, 59–61].

**Table 2:** Characteristics of included studies

References	Diagnostic criteria^1^/Indication	Treatment conditions	Age (mean, range/SD) (years)	Sample (Male)	Rating Scale	Treatment duration (weeks)	Design
**Alpha-2 adrenergic agonist agent**							
Scahill, 2001	DSM/ADHD+TD	Placebo Guanfacine	7-15 10.4±2.01	34(31) C:17 T:17	YGTSS	8	RCT
Cummings, 2002	DSM-IV/TS	Placebo Guanfacine	6-16 10.4±2.3	24(20) C:12 T:12	YGTSS	4	RCT
Goetz, 1987	DSM-III/TS	Placebo Clonidine	8-62	30(23) C:30 T:30	NR	12	Crossover
Leckman, 1991	DSM-III/TS	Placebo Clonidine	7-48	40(32) C:19 T:21	CGI	12	RCT
Du, 2008	CCMD-3/TD	Placebo Clonidine	6-18 C:10.15±2.82 T:9.89±2.77	437(366) C:111 T:326	NR	4	RCT
Singer, 1994	DSM-III-R/TS+ADHD	Placebo Clonidine, Desipramine	2-11	34(31) C:34 T1:34 T2:34	NR	6	RCT
Zhong, 2007	CCMD-III/TD	Placebo patch Clonidine patch	6-18	76(49) C:18 T:58	NR	4	Parallel RCT
Niederhofer, 2003	DSM-IV/ADHD+TD	Placebo Lofexidine	7-15 10.4±2.01	44(41) C:22 T:22	NR	8	RCT
Hedderick, 2009	DSM/TS	Levetiracetam Clonidine	8-30	10(7) C:10 T:10	YGTSS CGI	15	Crossover
**Analgesic**							
Kurlan, 1991	DSM-III-R/TS	Placebo Naltrexone, Propoxyphene	33±10	10(8) C:3 T1: 3 T2: 4	TSGS	6	RCT
**Anticonvulsant**							
Smith-hicks, 2007	DSM-IV/TS	Placebo Levetiracetam	8-16 12.2±2.3	22(21) C:22 T:20	YGTSS CGI	10	Crossover
Jankovic, 2010	DSM-IV/TS	Placebo Topiramate	7-65 C:14.1±8.35 T:18.8±10.93	29(26) T:15 C:14	YGTSS	10	RCT
**Antidepressant**							
Scahill, 1997	DSM-III-R/TS	Placebo Fluoxetine	8-33	14(9) C:10 T:10	YGTSS	20	Crossover
George, 1993	DSM-III-R/TS	Sulpiride Fluvoxamine	C:29.6±2.9 T:28.3±3.2	11(8) C:11 T:11	YGTSS	18	Crossover
Spencer, 2002	DSM-IV-R/ADHD+TD	Placebo Desipramine	5-17 C:11.3± 3.0 T:10.6 ± 2.4	41(34) C:20 T:21	YGTSS	6	RCT
Feigin, 1995	DSM-IIIR/ADHD+TS	Placebo Deprenyl	7-16	24(21) T:24 C:24	YGTSS	8	Crossover
**Antipsychotic agent**							
Shapiro, 1989	DSM-III/TS	Placebo Haloperidol, Pimozide	8-65 21.1±11.0	57(44) C:19 T1:18 T2:20	TSSS	6	Crossover
Sallee, 1997	DSM-III-R/TS	Placebo Haloperidol, Pimozide	7-16 10.2±2.5	22(17) C:22 T1:22 T2:22	TSGS	24	Crossover
Shapirc, 1984	DSM-III/TS	Placebo Pimozide	11-53 24.65±2.71	20(13) C:20 T:20	NR	14	Crossover
Sallee, 1999	DSM-IV/TS	Placebo Ziprasidone	7-16 C:11.3 (7-14) T:11.8 (8-16)	28(22) C:12 T:16	YGTSS CGI	8	RCT
Scahill, 2003	DSM-IV/TS	Placebo Risperidone	6-62 19.7±17.01	34(30) C:18 T:16	YGTSS	8	RCT
Dion, 2002	DSM-IV-R/TS	Placebo Risperidone	14-49 C:31( 17–49) T:33(14–45)	46(36) C:23 T:23	CGI TSSS	8	RCT
Gaffney, 2002	DSM-III-R/TS	Clonidine Risperidone	7-17 T:10.4±2.7 C:12.1±3.0	21(19) C:12 T:9	YGTSS	8	RCT
Gilbert, 2004	DSM-IV-TR/TD	Pimozide Risperidone	7-17 11±2.5	19(15) C:18 T:18	YGTSS CGI	4	Crossover
Yoo, 2013	DSM-IV/TD	Placebo Aripiprazole	6-18 C:11.0±25 T:10.9 ±3.0	61(53) C:29 T:32	YGTSS	10	RCT
Ghanizadeh, 2014	DSM-IV/TD	Risperidone Aripiprazole	6-18	60 C:29 T:31	YGTSS	8	RCT
Eggers, 1988	ICD-10/TS	Placebo Tiapride	7-18	17(25) C:17 T:17	NR	26	Crossover
Onofrj, 2000	Unclear/TS	Pimozide Olanzapine	19-40	4(4) C1:4 T1:4	TSGS	52	Crossover
**Cannabis**							
Muller-vahl,2002	DSM-III R/TS	Placebo Delta 9-tetrahy-drocannabinol	18-66	12(11) C:6 T:6	YGTSS TSGS	4	RCT
**CNS stimulant**							
Gadow, 1995	DSM-III-R/ADHA+TD	Placebo Methylphenidate	8 10 month	34(31) C:8 T1:9 T2:8 T3:9	YGTSS	2	RCT
Gadow, 1999	DSM-III-R/ADHD+TS	Placebo Methylphenidate	6.1-11.9	34(31) C:17 T:17	YGTSS CGI	2	RCT
Gadow, 2007	DSM/ADHA+TD	Placebo Methylphenidate	6-12 8.95±1.4	71(57) C:17 T1:17 T2:18 T3:18	YGTSS	2	RCT
Castellanos,1997	DSM-III-R/ADHD+TS	Placebo Methylphenidate, Dextroamphetamine	9.4 ±2.0	12 C:10 T1:10 T2:10	YGTSS	9	Crossover
Kurlan 2002	DSM-IV/ADHD+ TS	Placebo Methylphenidate, Clonidine, Methylphenidate + Clonidine	7-14	136(116) T1:37 T2:34 T3:33 C:32	YGTSS	16	RCT
**Cholinoceptor blocking drugs**							
Silver, 2001 a	DSM-IV/TS	Placebo Mecamylamine	8-17	61(55) C:32 T:29	NR	8	RCT
**Dopaminergic agent**							
Gilbert, 2003	DSM/TS, CTDs and ADHD	Placebo Pergolide	7-17 C:10.4±2.2 T:11.5 ±.9	51(77) C:15 T:36	YGTSS	8	RCT
Gilbert, 2000	DSM-IV/TS	Placebo Pergolide	7-17	24 (15) C:19 T:19	YGTSS	16	Crossover
Kurlan,2012	DSM-IV/TS	Placebo Pramipexole	6-17	63 C:20 T:43	YGTSS	6	RCT
Goetz,1994	DSM-III-R/TS	Placebo Talipexole	19-63	13(13) C:8 T:8	NR	24	Crossover
**Gamma-aminobutyric acid-B receptor agonist**							
Singer, 2001	DSM-IV/TS	Placebo Baclofen	8-14	10(7) C:10 T:9	CGI	10	RCT
**Glutamate agonist and antagonist**							
Lemmon, 2015	TS was defined by the TS Classification Study Group	Placebo D-serine, Riluzole	9-18	24(21) C:5 T1:10 T2:9	YGTSS	8	RCT
Bloch, 2016	DSM-IV/TS+TCD	Placebo N-Acetylcysteine	8-17	31(24) C:14 T:17	YGTSS CGI	12	RCT
**Selective norepinephrine reuptake inhibitor**							
Allen, 2005	DSM-IV/ADHD+TD	Placebo Atomoxetine	7-17 11.2± 2.5	148 (131) C:76 T:72	YGTSS CGI	18	RCT
Spencer, 2008	DSM-IV/ADHD+ TS	Placebo Atomoxetine	7-17 11.2 ± 2.4	117(102) C:56 T:61	YGTSS CGI	18	RCT
**Smoking cessation agent**							
Silver, 2001 b	DSM-IV/TD	Placebo Nicotine patch	T:10.5±1.8 C:11.7±2.6	70(63) C:35 T:35	YGTSS	4.71	RCT
**Smoking cessation agent plus Antipsychotic drugs**							
Mcconville 1992	DSM-III-R/TS	Placebo Nicotine patch plus haloperidol, Nicotine patch	8-46	19(16) C:5 T1:10 T2:9	YGTSS CGI	NR	RCT
**Traditional Chinese Medicine**							
Zhao, 2010	DSM-IV-TR/TS	Placebo Ningdong Granule	7-18 C:12.50±2.87 T:11.95±2.93	64(57) C:31 T:33	YGTSS	8	RCT
Zheng, 2016	DSM-IV/TS	Placebo Tiapride, 5-Ling Granule	5–18 9.8 ±3.0	603(511) C:117 T1:123 T2:363	YGTSS	8	RCT
Li, 2009	DSM-IV/TS	Haloperidol Ningdong Granule plus Haloperidol	C:9.60±2.95 T:9.59±3.00	90(70) C:30 T:60	YGTSS	25.71	RCT
Wu, 2010	ICD-10, TCM/TD	Haloperidol plus Trihexyphenidyl. Qufeng Zhidong Recipe	C:6.93±2.64 T:7.11±3.67	61(51) C:30 T:31	YGTSS	24	RCT
Wu, 2009	ICD-10, DSM-IV/TD	Haloperidol plus Trihexyphenidyl Qufeng Zhidong Recipe	C:9.10±1.13 T:9.70±2.01	81(66) C:40 T:41	YGTSS	24	RCT
**5HT3-receptor antagonists**							
Toran, 2005	DSM-IV/TS	Placebo Ondansetron	12-46 21.7±9.14	30(20) C:15 T:15	YGTSS TSGS	3	RCT
Nicolson, 2005	DSM-IV-TR/TS	Placebo Metoclopramide	7-18 T:12.4 ± 2.1 C:11.4 ± 3.1	27 C:13 T:14	YGTSS CGI	8	RCT

Note:

1. Diagnose:

DSM-IV: Diagnostic and Statistical Manual of Mental Disorders-IV

ICD-10: The International Statistical Classification of Diseases and Related Health Problems 10th Revision

DSM-III R: Diagnostic and Statistical Manual of Mental Disorders-III revision

The definition of TS Classification Study Group: onset before 18 years, multiple involuntary motor tics, one or more vocal tics, a waxing and waning course, the gradual replacement of old symptoms with new ones, the presence of tics for more than one year, the absence of other medical explanations for tics, and the observation of tics by a reliable examiner ADHD: attention deficit hyperactivity disorder

### Risk of bias

Risk of bias were showed in Table 3. The methodology qualities of randomisation and allocation concealment were less satisfactory. There were merely 15 reports (28%) which clearly described the generation of random sequence and 11 reports (20.8%) which described allocation concealment. Similarly, bindings of participant and outcome assessment were unspecified, which made unclear risk in 37 studies (64.2%) for binding of participant and in 43 studies (81.1%) for binding of outcome assessment. And none of the studies were judged to be prone to high risk of bias. The risk of bias regarding incomplete outcome data were high in 5 reports (9.4%) due to high rate of dropout with little explanation and low in 26 reports (49%). Selective report was not identified in any of the included articles. Finally, only 2 articles (3.8%) were found to be associated with other source of bias owning to obvious unbalance of baseline.

**Table 3:** Risk of bias in studies

Study ID	Random sequence generation	Allocation concealment	Blinding of participants	Blinding of outcome assessment	Incomplete outcome data	Selective reporting	Other sources of bias
Scahill, 2001	U	U	L	U	U	U	L
Cummings, 2002	U	L	L	U	U	U	L
Goetz,1987	U	U	U	U	L	U	U
Leckman,1991	U	U	U	U	H	U	L
Du,2008	L	U	L	L	U	U	L
Singer,1994	U	U	U	U	L	U	U
Zhong,2007	L	U	U	L	U	U	L
Niederhofer,2003	U	U	U	L	U	U	U
Hedderick, 2009	U	U	L	L	L	U	L
Kurlan,1991	U	U	U	U	U	U	U
Smith-hicks,2007	L	U	U	U	L	U	U
Jankovic, 2010	L	U	U	U	L	U	U
Scahill,1997	U	U	L	U	L	U	L
George,1993	U	U	U	L	L	U	U
Spencer,2002	U	L	U	U	U	U	L
Feigin, 1995	L	U	U	U	U	U	L
Shapiro, 1989	U	U	U	U	L	L	U
Sallee, 1997	U	L	U	U	L	U	U
Shapirc,1984	U	U	U	U	L	L	U
Sallee, 1999	U	U	U	U	U	H	U
Scahill, 2003	L	L	U	U	L	U	U
Dion, 2002	U	U	U	U	L	L	U
Gaffney,2002	U	U	U	U	L	L	U
Gilbert,2004	L	L	L	L	L	L	U
Yoo,2013	U	U	U	U	L	U	L
Ghanizadeh,2014	L	U	L	L	L	U	L
Eggers,1988	U	U	U	U	U	U	L
Onofrj, 2000	L	U	U	U	L	L	U
Muller-vahl,2002	U	L	L	L	L	U	U
Gadow,1995	U	U	U	U	U	U	L
Gadow,1999	U	U	U	U	U	U	L
Gadow,2007	U	L	U	U	U	U	L
Castellanos,1997	U	U	U	U	U	U	L
Kurlan,2002	L	L	L	U	L	U	L
Silver, 2001	U	U	U	U	U	U	L
Gilbert, 2003	U	U	L	U	H	U	U
Gilbert, 2000	L	L	L	U	L	U	L
Kurlan,2012	U	U	U	U	H	U	L
Goetz,1994	U	U	U	L	U	U	U
Singer, 2001	L	U	L	U	U	U	H
Lemmon,2015	L	U	L	U	U	U	U
Bloch,2016	U	L	L	L	U	U	U
Allen,2005	L	U	U	U	L	U	L
Spencer,2008	U	U	U	U	H	U	L
Silver,2001	L	L	L	U	H	U	L
Mcconville,1992	U	U	U	U	L	U	L
Zhao,2010	U	L	L	L	L	L	U
Zheng,2016	U	U	L	U	L	L	L
Li, 2009	L	U	U	U	L	L	U
Wu,2010	U	U	U	U	U	U	U
Wu,2009	L	U	U	U	U	L	U
Toran,2005	U	U	U	U	U	U	H
Nicolson,2005	U	U	U	U	L	U	L

Note: H: high risk; L:low risk; U:unclear

### Meta-analytic results

Forty-one studies of the included 53 studies involving 34 pharmacological interventions reported one of the outcome measures and were brought into meta-analyses, among which 33 studies (29 types of intervention) compared pharmacotherapies with placebo and 11 studies (16 types of intervention) compared pharmacotherapies with each other (3 studies were placebo controlled as well).

#### Placebo controlled comparisons

Table 4 to Table 7 demonstrated the efficacy of pharmacotherapies compared to placebo in each outcome measure.

**Table 4:** Effect sizes of pharmacological interventions compared with placebo using the outcome scale of YGTSS for patients with Tourette syndrome

Concomitant Drug/Study	YGTSS		
Author (Year)	N^1^	Age^2^	WMD
Alpha-2 adrenergic agonist agent			
Guanfacine	2	a	**-4.596(-8.798,-0.393)**
Scahill,2001	1	a	**-4.500(-8.939,-0.061)**
Cummings,2002	1	a	-5.420(-18.461,7.621)
Anticonvulsant			
Levetiracetam			
Smith-hicks,2007	1	a	0.050(-16.175,16.275)
Topiramate			
Jankovic, 2010	1	b	**-9.290(-16.697,-1.883)**
Antidepressant			
Desipramine			
Spencer,2002	1	a	**-16.000(-27.130,-4.870)**
Fluoxetine			
Scahill,1997	1	b	1.100(-6.325,8.525)
Antipsychotic agent			
Atypical Antipsychotic agent	4		
Ziprasidone			
Sallee,1999	1	a	**-6.900(-11.234,-2.566)**
Risperidone			
Scahill,2003	1	b	**-6.400(-11.059,-1.741)**
		a	**-7.100(-12.276,-1.924)**
Aripiprazole			
Yoo,2013	1	a	**-5.100(-9.178,-1.022)**
Tiapride			
Zheng,2016	1	a	**-11.700(-15.101,-8.299)**
Cannabis			
Delta-9-tetrahydrocannabinol			
Muller-vahl,2002	1	b	-6.500(-19.174,6.174)
CNS stimulant			
Methylphenidate	7	a	0.035(-4.442,4.512)
0.1 mg/kg			-0.759(-9.270,7.752)
Gadow,1995	1	a	1.000(-14.637,16.637)
Gadow,2007	1	a	-1.500(-11.645,8.645)
0.3 mg/kg			1.263(-7.307,9.832)
Gadow,1995	1	a	3.600(-12.907,20.107)
Gadow,2007	1	a	0.400(-9.627,10.427)
0.5 mg/kg			-0.706(-9.119,7.707)
Gadow,1995	1	a	0.800(-15.021,16.621)
Gadow,2007	1	a	-1.300(-11.234,8.634)
Mixed dosage(0.1,0.3,0.5 mg/kg)			
Gadow,1999	1	a	0.600(-10.347,11.547)
Dopaminergic agent			
Pergolide	2	a	**-13.167(-20.553,-5.781)**
Gilbert,2003	1	a	-8.800(-18.761,1.161)
Gilbert,2000	1	a	**-18.500(-29.508,-7.492)**
Pramipexole			
Kurlan,2012	1	a	-0.150(-2.277,1.977)
Glutamate agonist			
D-serine			
Lemmon,2015	1	a	-2.600(-19.985,14.785)
N-Acetylcysteine			
Bloch,2016	1	a	2.200(-2.830,7.230)
Glutamate antagonist			
Riluzole			
Lemmon,2015	1	a	-4.100(-23.452,15.252)
Selective norepinephrine reuptake inhibitor			
Atomoxetine	2	a	**-2.767(-4.649,-0.882)**
Allen,2005	1	a	-2.500(-5.023,0.023)
Spencer,2008	1	a	**-3.100(-5.931,-0.269)**
Smoking cessation agent			
Nicotine patch	2		**-7.018(-8.252,-5.783)**
Mcconville,1992	1	b	-0.300(-11.511,10.911)
Silver, 2001	1	a	**-7.100(-8.342,-5.858)**
Smoking cessation agent plus Antipsychotic drugs			
Nicotine patch plus haloperidol			
Mcconville,1992	1	b	-6.400(-16.549,3.749)
Traditional Chinese medicine			
NingDong Granule			
Zhao,2010	1	a	**-7.100(-10.430,-3.770)**
5-Ling Granule			
Zheng,2016	1	a	**-11.300(-14.208,-8.392)**
5HT3-receptor antagonists			
Ondansetron			
Toran,2005	1	b	-2.000(-9.203,5.203)
Metoclopramide			
Nicolson,2005	1	a	**-5.900(-10.147,-1.653)**

Note: Significant results are in bold.

1: N:number of studies;

2: a represented that the subjects included in the study were children; b represented that the subjects included in the study were mixed participation (children and adult).

**Table 5:** Effect sizes of pharmacological interventions compared with placebo using the outcome scale of CGI for patients with Tourette syndrome

Concomitant Drug/Study (N)	CGI		
Author (Year)	N^1^	Age^2^	WMD
Alpha-2 adrenergic agonist agent			
Clonidine			
Leckman,1991	1	b	**-0.600(-0.996,-0.204)**
Anticonvulsant			
Levetiracetam			
Smith-hicks,2007	1	a	0.090(-0.572,0.752)
Antipsychotic agent			
Atypical Antipsychotic agent			
Ziprasidone			
Sallee,1999	1	a	-0.700(-1.407,0.007)
Risperidone			
Dion,2002	1	b	**-0.650(-1.207,-0.093)**
CNS stimulant			
Methylphenidate			
Mixed dosage(0.1,0.3,0.5 mg/kg)			
Gadow,1999	1	a	0.000(-0.410,0.410)
Gamma-aminobutyric acid-B receptor agonist			
Baclofen			
Singer,2001	1	a	**-0.900(-1.497,-0.303)**
Glutamate agonist			
N-Acetylcysteine			
Bloch,2016	1	a	0.100(-0.622,0.822)
Selective norepinephrine reuptake inhibitor			
Atomoxetine	2	a	**-0.644(-0.910,-0.378)**
Allen,2005	1	a	**-0.600(-0.957,-0.243)**
Spencer,2008	1	a	**-0.700(-1.099,-0.301)**
Smoking cessation agent plus Antipsychotic drugs			
Nicotine patch plus haloperidol			
Mcconville,1992	1	b	-0.800(-1.901,0.301)
5HT3-receptor antagonists			
Metoclopramide			
Nicolson,2005	1	a	**-1.000(-1.639,-0.361)**

Note: Significant results are in bold.

1: N:number of studies;

2: a represented that the subjects included in the study were children; b represented that the subjects included in the study were mixed participation (children and adult).

**Table 6:** Effect sizes of pharmacological interventions compared with placebo using the outcome scale of TSGS for patients with Tourette syndrome

Concomitant Drug/Study (N)	TSGS		
Author (Year)	N^1^	Age^2^	WMD
Analgesic			
Naltrexone			
Kurlan,1991	1	b	-0.100(-6.426,6.226)
Propoxyphene			
Kurlan,1991	1	b	**-8.700(-14.711,-2.689)**
Antipsychotic agent			
Typical Antipsychotic agent			
Haloperidol			
Sallee,1997	1	a	-6.100(-15.361,3.161)
Pimozide			
Sallee,1997	1	a	**-9.700(-18.436,-0.964)**
Cannabis			
Delta-9-tetrahydrocannabinol			
Muller-vahl,2002	1	b	-6.500(-15.652,2.652)
5HT3-receptor antagonists			
Ondansetron			
Toran,2005	1	b	-2.680(-16.742,11.382)

Note: Significant results are in bold.

1: N:number of studies;

2: a represented that the subjects included in the study were children; b represented that the subjects included in the study were mixed participation (children and adult).

**Table 7:** Effect sizes of pharmacological interventions compared with placebo using the outcome scale of TSSS for patients with Tourette syndrome

Concomitant Drug/Study (N)	TSSS		
Author (Year)	N1	Age2	WMD
Antipsychotic agent			
Typical Antipsychotic agent			
Haloperidol			
Shapiro,1989	1	b	**-1.700(-3.006,-0.394)**
Pimozide			
Shapiro,1989	1	b	-0.400(-1.952,1.152)
Atypical Antipsychotic agent			
Risperidone			
Dion,2002	1	b	**-1.070(-2.092,-0.048)**

Note: Significant results are in bold.

1: N:number of studies;

2: a represented that the subjects included in the study were children; b represented that the subjects included in the study were mixed participation (children and adult).

Twenty-two interventions (from 33 studies) were more efficacious than placebo. It's worth noting that among the above effective treatments, only 4 interventions including guanfacine, pergolide, atomoxetine, and nicotine patch incorporated two trials, while the rest of these merely incorporated one.

### Alpha-2 adrenergic agonist agents

**Figure 2:**
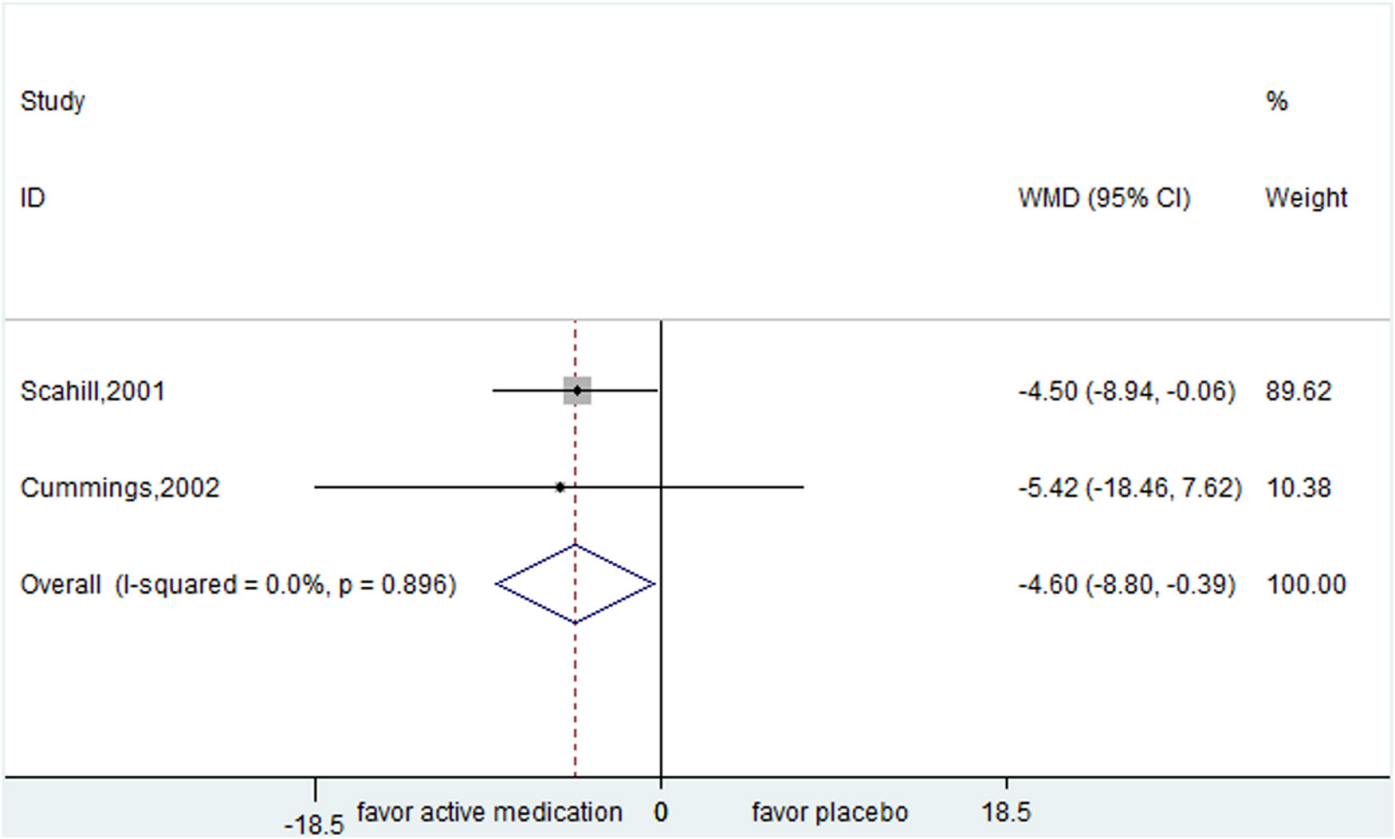
Efficacy of guanfacine compared with placebo for the treatment of tics in the outcome of Yale Global Tic Severity Scale

Three studies evaluated the efficacy of alpha-2 adrenergic agonist agents. Guanfacine from two trials showed positive effect for children suffering from tics (YGTSS:WMD= -4.596, 95% CI, -8.798- -0.393) (Figure 2). Clonidine was superior to placebo (CGI: WMD=-0.600, 95% CI, -0.996- -0.204) in one trial conducted in mixed patients.

### Analgesic

Evidences of two analgesics from 2 mixed patients’ studies showed opposite results. Propoxyphene was effective in suppressing tics (TSGS: WMD=-8.700, 95% CI, -14.711- -2.689), while naltrexone failed to improve tics (TSGS: WMD=-0.100, 95% CI, -6.426- 6.226).

### Anticonvulsants

Similarly, evidences of two anticonvulsants showed opposite results. The trial by Jankovic and colleagues discovered that topiramate might be effective in suppressing children tics (YGTSS: WMD=-9.290, 95% CI, -16.697- -1.883). Even though the range of age in this study was 7-65, mean age was 16.5 with the majority of participants being children. For levetiracetam, it was found to be lack of efficacy in children (YGTSS: WMD=0.050, 95% CI, -16.175- 16.275).

### Antidepressants

Likewise, for antidepressants, desipramine (tricyclic antidepressant) was more efficacious than placebo in children (YGTSS:WMD=-16.000, 95% CI,-27.130- -4.870). However, Fluoxetine, another kind of antidepressant (selective serotonin reuptake inhibitor), was found to be lack of efficacy in mixed participants (YGTSS: WMD=1.100, 95% CI, -6.325- 8.525).

### Antipsychotic agents

Nearly all antipsychotic agents yielded positive effect, and atypical antipsychotic agents covered more efficacious agents and outcome scales than typical antipsychotic agents. The following agents yielded positive effects: atypical antipsychotic agents (ziprasidone (YGTSS: WMD=-6.900, 95% CI,-11.234- -2.566); risperidone (YGTSS: WMD=-6.400, 95% CI, -11.059- -1.741; CGI: WMD=-0.650, 95% CI, -1.207- -0.093); aripiprazole (YGTSS: WMD=-5.100, 95% CI, -9.178- -1.022); tiapride (YGTSS: WMD=-11.700, 95% CI, -15.101- -8.299)) and typical antipsychotic agent (haloperidol (TSSS: WMD=-1.700, 95% CI, -3.006- -0.394); pimozide (TSGS: WMD=-9.700, 95% CI, -18.436- -0.964)), while the following agents yielded negative effects: ziprasidone in the outcome of CGI( WMD=-0.700, 95% CI, -1.407- 0.007), haloperidol in TSGS (WMD=-6.100, 95% CI, -15.361- 3.161), and pimozide in TSSS (WMD=-0.400, 95% CI, -1.952- 1.152). For studies conducted in mixed population, only the separate effect of risperidone in children was found (YGTSS: WMD=-7.100, 95% CI, -12.276- -1.924).

### Cannabis

Delta-9-tetrahydrocannabinol from one trial was not significantly different from placebo in the mixed participants (YGTSS: WMD=-6.500, 95% CI, -19.174- 6.174; TSGS: WMD=-6.500, 95% CI, -15.652- 2.652).

### CNS (central nervous system) stimulants

Evidences showed that methylphenidate might not exacerbate tics (YGTSS: WMD=0.035, 95% CI, -4.442- 4.512), no matter children receiving which kinds of therapeutic regimens, including 0.1 mg/kg (WMD=-0.759, 95% CI,-9.270- 7.752), 0.3 mg/kg (WMD=1.263, 95% CI,-7.307- 9.832), and 0.5 mg/kg (WMD=-0.706, 95% CI, -9.119- 7.707). There was no sign of heterogeneity from statistical test (I^2^ = 0%) among each regimen.

### Dopaminergic agent

**Figure 3:**
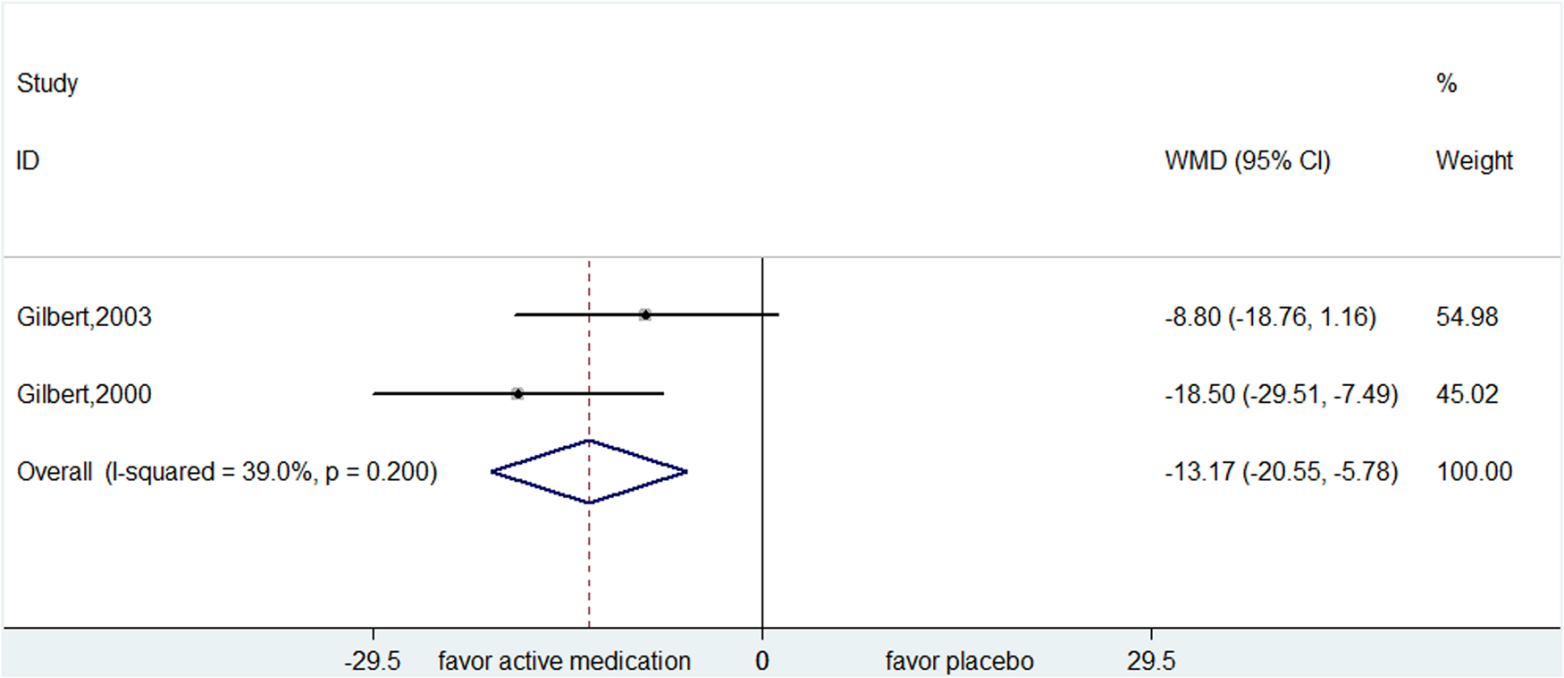
Efficacy of pergolide compared with placebo for the treatment of tics in the outcome of Yale Global Tic Severity Scale

Results of the pergolide and pramipexole were inconsistent. Pergolide was superior to placebo by pooling two trials

(YGTSS: WMD=-13.167, 95% CI, -20.553- -5.781) (Figure 3), while pramipexole was found to be lack of efficacy (YGTSS: WMD=:-0.150, 95% CI, -2.277- 1.977).

### Gamma-aminobutyric acidB receptor agonist

Baclofen was superior to placebo from one trial (CGI: WMD=-0.900, 95% CI, -1.497- -0.303).

### Glutamate agonist and antagonist

As to glutamate modulators (D-serine, N-Acetylcysteine and riluzole), each in one trial, they were all not significantly different from placebo in the measure of YGTSS (D-serine: WMD= -2.600, 95% CI, -19.985- 14.785; N-Acetylcysteine: WMD=2.200, 95% CI, -2.830- 7.230; riluzole WMD=-4.100, 95% CI, -23.452- 15.252).

### Selective norepinephrine reuptake inhibitor

**Figure 4:**
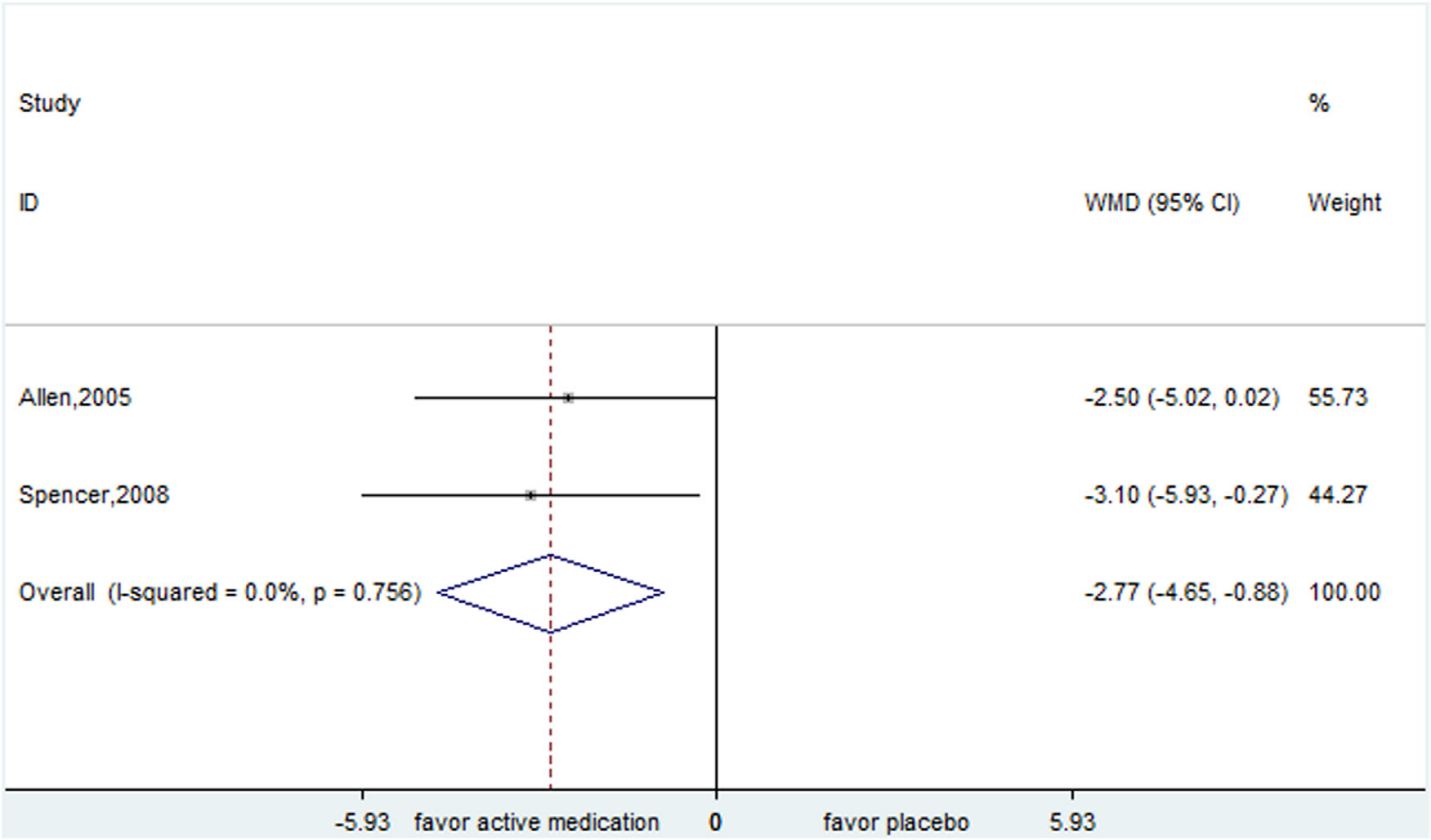
Efficacy of atomoxetine compared with placebo for the treatment of tics in the outcome of Yale Global Tic Severity Scale

**Figure 5:**
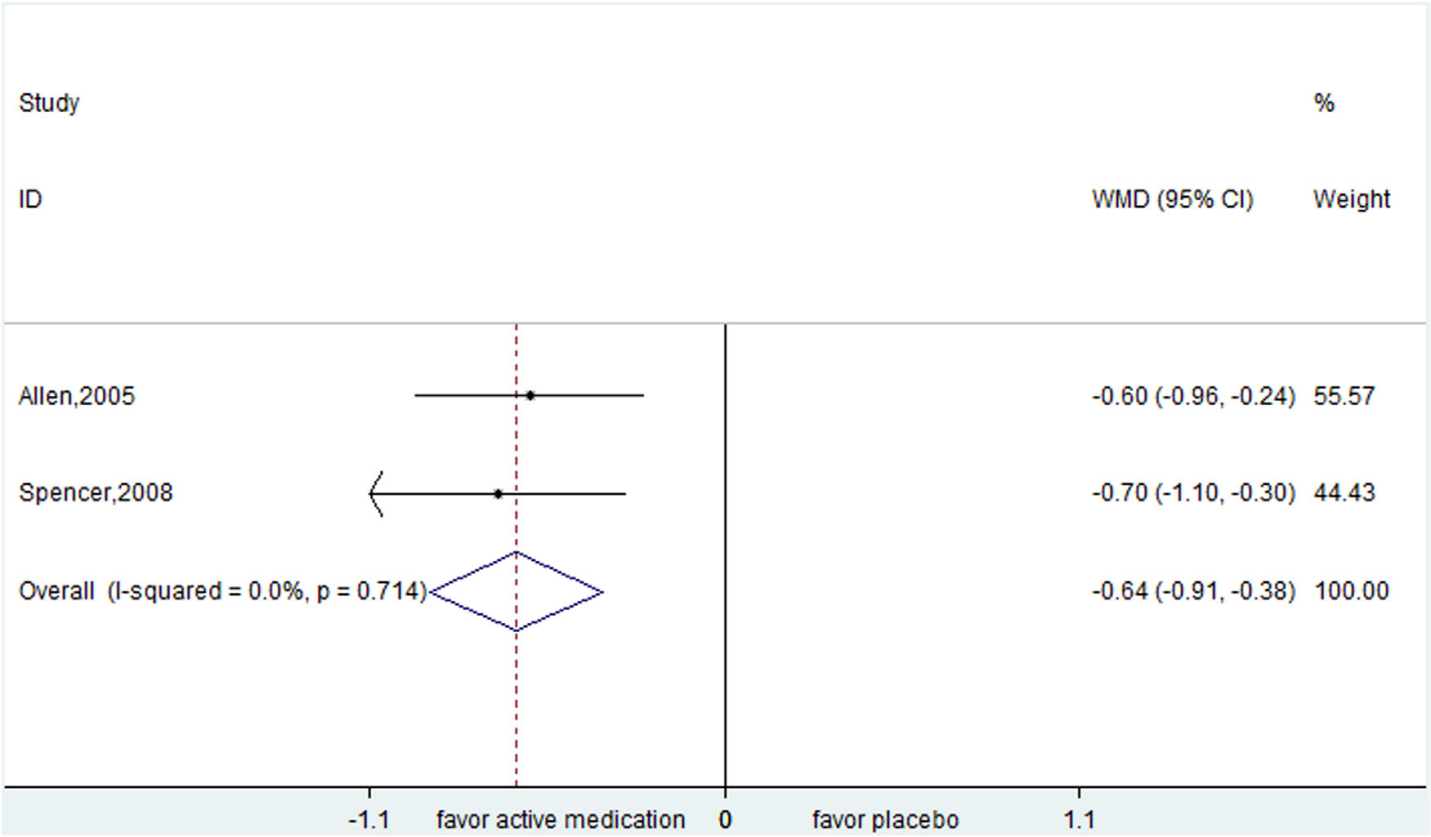
Efficacy of atomoxetine compared with placebo for the treatment of tics in the outcome of Clinical Global Impression Scale

Atomoxetine from two trials was superior to placebo in children (YGTSS: WMD=-2.767, 95% CI, -4.649- -0.882; CGI: WMD= -0.644, 95% CI, -0.910- -0.378) (Figure 4 and 5).

### Smoking cessation agent

**Figure 6:**
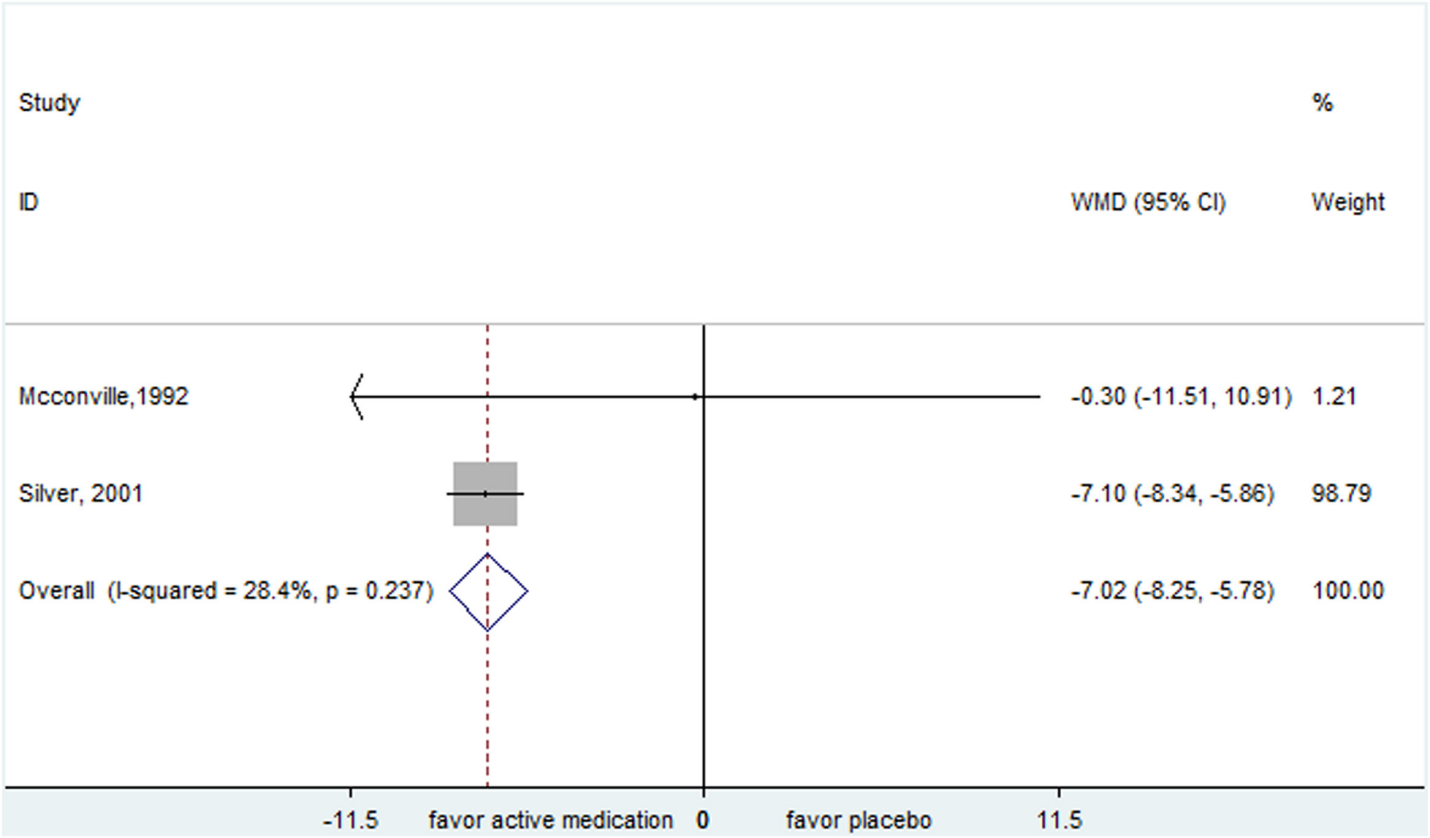
Efficacy of nicotine patch compared with placebo for the treatment of tics in the outcome of Yale Global Tic Severity Scale

For nicotine patch, the pooled result of YGTSS showed a positive effect (WMD=-7.018, 95% CI, -8.252- -5.783) (Figure 6). It's worth noting that Silver et al. reported a favorable effect of nicotine patch in children (YGTSS: WMD= -7.100, 95% CI,-8.342- -5.858), however, another trial by McConville et al. including patients with the mean age of 22 discovered that nicotine patch might not improve tics (YGTSS: WMD=-0.300, 95% CI, -11.511- 10.911). The explanation to these might be that the two studies included subjects with different age range. Moreover, McConville et al. also evaluated the effect of nicotine patch plus haloperidol compared to placebo and found that there was no significant difference between treatments in the scale of YGTSS (WMD=-6.400, 95% CI, -16.549- 3.749) and CGI (WMD=-0.800, 95% CI, -1.901- 0.301).

### Traditional Chinese medicine

Traditional Chinese Medicine (TCM) (NingDong Granule and 5-Ling Granule) all showed positive effects in the scale of YGTSS (NingDong Granule: WMD=-7.100, 95% CI, -10.430- -3.770; 5-Ling Granule: WMD=-11.300, 95% CI, -14.208- -8.392).

### 5HT3-receptor antagonists

The study of ondansetron was conducted by Toran et al. in the mixed patients (mean age was 21.7±9.14) and failed to show a positive effect (YGTSS: WMD= -2.000, 95% CI, -9.203- 5.203; TSGS: WMD=-2.680, 95% CI, -16.742- 11.382). Conversely, metoclopramide tested in a children trial was superior to placebo (YGTSS: WMD=-5.900, 95% CI, -10.147- -1.653, CGI: WMD=-1.000, 95% CI, -1.639- -0.361).

#### Head-to-head comparisons

Table 8 to Table 10 demonstrated the efficacy of pharmacotherapies compared to each other in each outcome measure.

**Table 8:** Effect sizes of pharmacological interventions from head-to head trials using the outcome scale of YGTSS for patients with Tourette syndrome

Concomitant Drug/Study	YGTSS		
Author (Year)	N^1^	Age^2^	WMD
NingDong Granule plus Haloperidol VS Haloperidol			
Li,2009	1	a	**-4.260(-7.284,-1.236)**
Qufeng Zhidong Recipe VS Haloperidol plus Trihexyphenidyl	2	a	**-16.886(-18.073,-15.700)**
Wu,2009	1		**-14.340(-16.194,-12.486)**
Wu,2010	1		**-18.650(-20.193,-17.107)**
5-Ling Granule VS Tiapride			
Zheng,2016	1	a	0.400(-2.304,3.104)
Clonidine VS Levetiracetam			
Hedderick, 2009	1	b	-2.000(-15.455,11.455)
Clonidine VS Risperidone			
Gaffney,2002	1	a	-2.900(-15.142,9.342)
Aripiprazole VS Risperidone			
Ghanizadeh,2014	1	a	-5.300(-11.430,0.830)
Risperidone VS Pimozide			
Gilbert,2003 b	1	a	-9.000(-23.949,5.949)
Fluvoxamine VS Sulpiride			
George,1993	1	b	5.000(-19.459,29.459)
D-serine VS Riluzole			
Yoo,2013	1	a	1.500(-16.106,19.106)

Note: Significant results are in bold.

1: N:number of studies;

2: a represented that the subjects included in the study were children; b represented that the subjects included in the study were mixed participation (children and adult).

**Table 9:** Effect sizes of pharmacological interventions from head-to head trials using the outcome scale of CGI for patients with Tourette syndrome

Concomitant Drug/Study (N)	CGI		
Author (Year)	N^1^	Age^2^	WMD
Clonidine VS Levetiracetam			
Hedderick, 2009	1	b	-0.100(-0.675,0.475)
Risperidone VS Pimozide			
Gilbert,2003 b	1	a	-1.000(-2.076,0.076)

Note: Significant results are in bold.

1: N:number of studies;

2: a represented that the subjects included in the study were children; b represented that the subjects included in the study were mixed participation (children and adult).

**Table 10:** Effect sizes of pharmacological interventions from head-to head trials using the outcome scale of TSGS for patients with Tourette syndrome

Concomitant Drug/Study	TSGS		
Author (Year)	N^1^	Age^2^	WMD
Olanzapine VS Pimozide			
Onofrj,2000	1	b	**-13.000(-15.504,-10.496)**

Note: Significant results are in bold.

1: N:number of studies;

2: a represented that the subjects included in the study were children; b represented that the subjects included in the study were mixed participation (children and adult).

Three interventions (from 11 studies) out of 16 interventions including NingDong Granule plus haloperidol, Qufeng Zhidong Recipe, and olanzapine showed significant positive effects compared to another interventions including haloperidol, haloperidol plus trihexyphenidyl, and pimozide, respectively. It's worth noting that among the above comparisons only one (Qufeng Zhidong Recipe versus haloperidol plus trihexyphenidyl) incorporated two trials, while the rest merely incorporated one.

**Figure 7:**
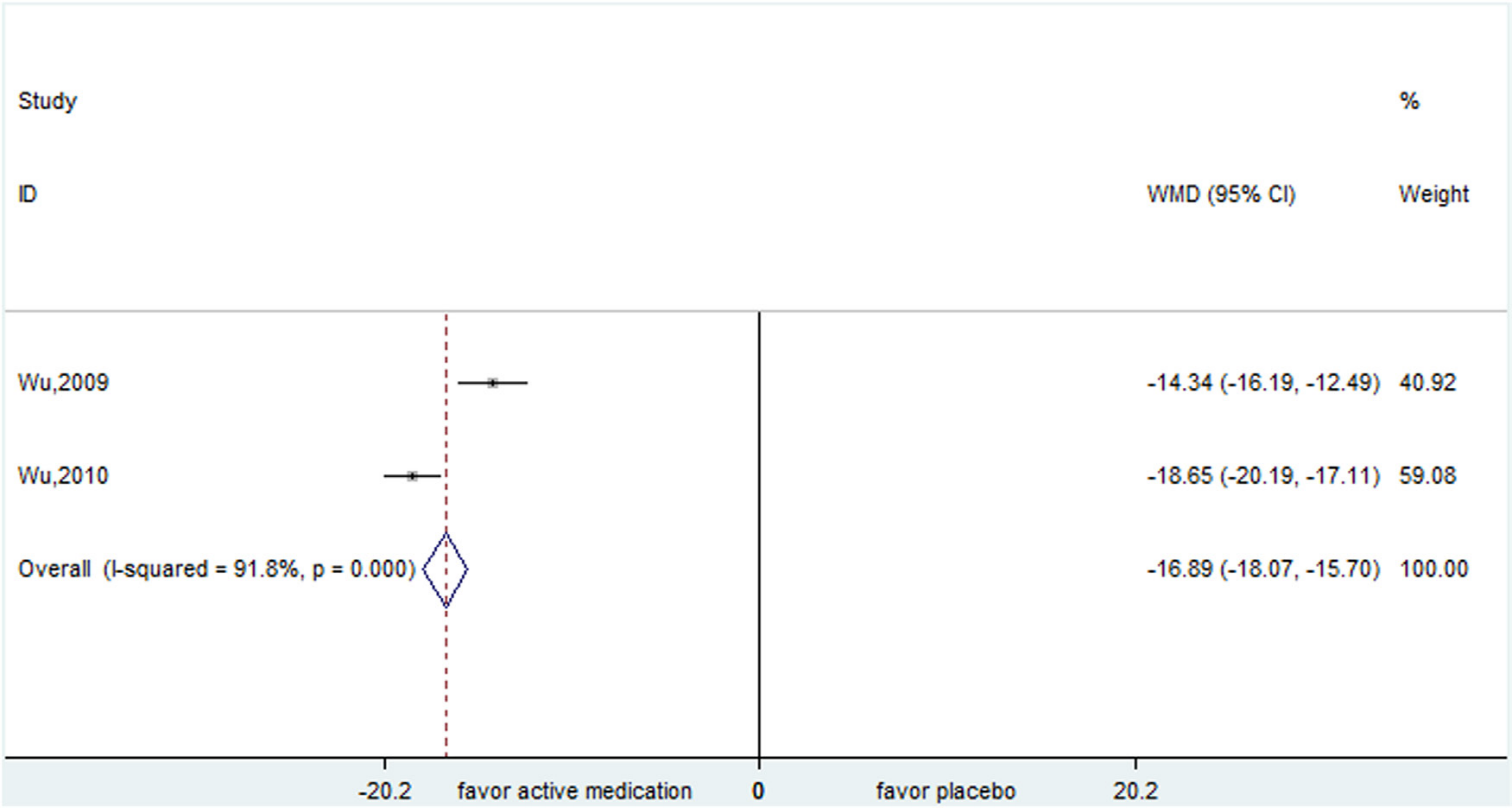
Efficacy of Qufeng Zhidong Recipe compared with Haloperidol plus trihexyphenidyl for the treatment of tics in the outcome of Yale Global Tic Severity Scale

Four studies tested the efficacy of TCM alone or plus western medicines compared to western medicines. One trial evaluated the comparison of NingDong Granule plus haloperidol versus haloperidol. Two trials evaluated the comparison of Qufeng Zhidong Recipe versus haloperidol plus trihexyphenidyl. The above two comparisons all yielded significant differences in the measure of YGTSS (WMD=-4.260, 95% CI, -7.284- -1.236; WMD=-16.886, 95% CI, -18.073- -15.700 (Figure 7); respectively). Another study was about a larger multisite, double-blind randomized, placebo-controlled trial of 603 children randomized to 5-Ling Granule (N=363), tiapride (N=123) or placebo (N=117) for 8 weeks. This trial failed to indicate a significant positive effect of 5-Ling Granule compared to tiapride (WMD=0.400, 95% CI, -2.304- 3.104).

Three researches tested the different efficacy among diverse types of antipsychotic agents. Only olanzapine was found to be less effective than pimozide in the outcome of TSGS WMD=-13.000, 95% CI,-15.504- -10.496). Other comparisons, including aripiprazole versus risperidone and risperidone versus pimozide, yielded no significant differences.

Studies indicated that clonidine was not significant different from levetiracetam (YGTSS: WMD=-2.000, 95% CI,-15.455- 11.455, CGI: WMD=-0.100, 95% CI, -0.675- 0.475) and risperidone (YGTSS: WMD=-2.900, 95% CI, -15.142- 9.342). Similarly, there were no significant differences between fluvoxamine versus sulpiride (YGTSS: WMD=5.000, 95% CI, -19.459- 29.459) and D-serine versus riluzole (YGTSS: WMD=1.500, 95% CI, -16.106- 19.106).

### Adverse effect

Detailed adverse effects of pharmacological interventions were displayed in Supplementary Table (1-14).

Of note, antipsychotic agents, especially typical neuroleptics, were associated with severer adverse effects, including weight gain, akathisia, and acute dystonia, than alpha-2 adrenergic agonist agents. Weight gain was a concern for the typical neuroleptics.

### Sensitivity analyses and publication bias

None of the interventions included more than two studies in meta-analysis, so sensitivity analyses and publication bias were not assessed according to the recommendations of the Cochrane Handbook for Systematic Reviews of Interventions (www.cochranehandbook.org).

## DISCUSSION

Meta-analysis suggested that alpha-2 adrenergic agonist agents and atypical antipsychotic agents were effective in improving tics, which included the maximum number of trials. Although typical antipsychotic agents were widely used in patients suffered from tics, certain evidences were scarce. Besides, according to head-to-head trials, there were no significant differences among the comparisons of antipsychotic agents and alpha-2 adrenergic agonist agents in efficacy, but the side effects among them were diverse. Antipsychotics agents, especially typical antipsychotic agents, were associated with severer side-effects than alpha-2 adrenergic agonist agents. Similar to several clinical guidelines [10, 23], typical and atypical neuroleptics were recommended with several evidences, but side effects restricted their use as first-line options.

In addition, for agents (e.g. CNS stimulants and atomoxetine) primarily used to manage comorbid tics and ADHD, evidences suggested that CNS stimulants might not exacerbate tics and atomoxetine might improve tics. For agents (e.g. fluoxetine) primarily used to manage comorbid tics and obsessive compulsive disorder/obsessive compulsive symptom, evidences suggested that fluoxetine might not exacerbate tics. These results were in close agreement with relevant systematic reviews and meta-analyses which suggested that CNS stimulants might not deteriorate tics [24, 80]. Nonetheless, clinical reports found that tics might become worse in patients undergoing high doses of stimulants [59].

In this meta-analysis, another significant discovery for clinical practice was the positive efficacy of TCM. Both of NingDong Granule and 5-Ling Granule might be more efficacious than placebo. NingDong Granule plus haloperidol might be more efficacious than haloperidol and Qufeng Zhidong Recipe might be more efficacious than haloperidol plus trihexyphenidyl. Unlike western medications, these three TCM (NingDong Granule, 5-Ling Granule, and Qufeng Zhidong Recipe) were specifically developed for tics. Traditional Chinese medicine believed that tics belonged to the sort of chronic infantile convulsion and hyperspasmia, and the major cause was yin-insufficiency in heart and liver [73, 81]. NingDong Granule, composed of eight different Chinese herbs and natural materials, could relieve convulsion and spasm by nourishing heart and liver yin [82]. Preclinical trials found that NingDong Granule improved the stereotypical behaviour of apomorphine-induced tics rats, an animal model of tics, by suppressing the dopamine system [28, 83]. 5-Ling Granule, a patented polyherbal product manufactured from 11 herbal materials, suppressed hyperactivity and tranquilize fidgetiness [74, 84]. Preclinical trials found that 5-Ling Granule suppressed head twitching and stereotyped movement in rat model induced by 3, 3’-iminodipropionitrile (IDPN), a synthetic neurotoxin. It also suppressed the stimulant amphetamine (AMP)-induced hyperactivity and irritability in mice. It's worth noting that different from haloperidol, a typical antipsychotic agent, 5-Ling Granule did not changed CNS excitability or spatial cognition [84]. Western medications for the management of tics were initially designed for other diseases, and accompanied by many adverse effects. For example, antipsychotic agents cut off dopamine receptors (act as dopamine antagonists), which generated the risk of extrapyramidal symptoms (EPS), dystonia, parkinsonism, and tardive dyskinesia. Traditional Chinese medicine also believed that the basic pathogenesis of tics was generally internal Lung-wind upsetting. Qufeng Zhidong Recipe was formulated by modifying the classic recipe for dispelling external wing [77].

However, firm conclusions were unable to draw due to the relatively tiny number of trials and findings needed to be replicated in more trials.

In general, the findings aligned with the review conducted by Craig Whittington et al [85]. Results showed that antipsychotic agents and alpha-2 adrenergic agonist agents demonstrated positive compared with placebo in improving tics. Different from their work which only included placebo-controlled trials, this review included head-to-head trials as well. Besides, this review included more comprehensive kinds of pharmacotherapies, such as TCM (NingDong Granule and 5-Ling Granule) and glutamate modulators (D-serine, N-Acetylcysteine and riluzole). The efficacy of different doses of methylphenidate were evaluated as well.

Furthermore, this results largely agreed with previous review by Chris Hollis et al.[86], despite this meta-analysis using various outcome measures. However, they only included medication with marketing authorisation in North America, Europe or Australasia, their results of pharmacotherapies were not comprehensive. Besides, they conducted a survey to capture qualitative and descriptive data on young people's experiences of treatment.

Concerning the review by Weisman et al. [87], it was too mechanistic to standardize the information of existing studies on pharmacotherapies for tics to perform a meta-analysis. They concentrated solely on the efficacy of FDA-approved antipsychotic agent or alpha-2 adrenergic agonists for treating tics, which failed to evaluate the side-effect profile and other kinds of pharmacological treatments. In terms of statistical analysis, although they performed a subgroup analysis by classifying studies according to comorbid ADHD condition and meta-regression to examine the relationship between efficacy and several natural variables, including trial duration, trial methodological quality, and percentage of subjects with TS, the number of studies included in each category was only five. The statistical power with such few studies was too low to detect meaningful results. So even though these factors possibly might influence the outcomes, relevant analyses were not conducted in this review.

A number of superiorities existed in this research. First, this review was reported based on PRISMA recommendations [88]. Second, in order to minimize error, three independent investigators were used in the part of literature screening, data extraction and risk of bias evaluation [89]. Third, in order to make a comprehensive assessment of pharmacological agents, all trials with subjects at any age suffering from tics were included in this systematic review. Fourth, this review not only included placebo controlled trials, but also assessed head-to-head trials, which were more comprehensive. Fifth and finally, the results with clear age of subjects were displayed.

However, several limitations needed to be considered when comprehending this meta-analysis.

First, despite the above clear results, the available studies of each intervention were few in number, which lacked sufficient statistical power for realistically evaluating the efficacy and publication bias. There was only one research about many treatments. Especially for head-to-head comparisons, the conclusions of no significant difference might not mean no difference in practice, which might be caused by few evidences with insufficient statistical power. Besides, the methodological quality of several included studies were not very well. As a consequence, conclusions were not completely certain and required repetition in more amount of populations. Second, the included trials evaluated short-term efficacy and safety in general, which meant that long-term outcomes were uncertain. Especially for side-effects, the reporting in controlled trials were less than desirable and a number of trials even did not recorded. Given that uncontrolled longer-term trials applying agents to tics and other disorders could provide more full-scale estimation, the overall assessment of adverse effects should take these studies into consideration. But these were beyond the range of this review, which made more reliable and practical adverse effects to be under-estimated. However, some important adverse effects were still detected in this review. By limiting to controlled trials, more dependable estimates of the percentage of subjects suffering from adverse events were able to be obtained. Third, although complications such as ADHD might impact the effect of alpha-2 agonists agents [87], the influence of complications on effect size was not measured, because the number of studies on each intervention was too small with inadequate statistical power to identify difference. What's more, meta-analytic approach is not the best tool to identify the influence of complications because the potential variability is usually correlated among trials. For instance, some subjects in trials with long-term duration tend to develop new complications in the period of research, which are very inconvenience to count in original trials and difficult to analyze in meta-analyses. Fourth, the effects of pharmacotherapies on motor and vocal tics separately were failed to be evaluated due to the inconsistent reporting among studies. Fifth, the outcome measures included in this review might be not very appropriate. For example, the quality of YGTSS to measure effects after 8 weeks is uncertainty, and CGI is a short inventory and not tic specific scale compared to the YGTSS. Last but not least, failing to identify unpublished trials might generated publication bias.

Future studies should include larger sample sizes to help minimize random error. Longer-term and head-to-head investigations will be required as well. What's more, about the data collection in original trial, recording some clinical confounders such as comorbidities would help comprehensively assess the efficacy of pharmacological strategies for patients suffering from tics.

## MATERIALS AND METHODS

### Search strategy

Our review was conducted and reported according to the PRISMA statement [90].

The literature retrieval was performed from 1976 to July 2015 (updated in Oct 2016) in Medline, Embase, The Cochrane Library, Chinese Biomedical Literature Database (CBM), China Knowledge Resource Integrated Database (CNKI), VIP Database, and Wanfang Database applying the following key words: tic or tics or tourette^*^. Citations of relevant studies were screened carefully for inclusion as well. Two reviewers independently screened the literatures referring to inclusion and exclusion criteria, and a third reviewer coordinated if they yielded disagreements.

### Inclusion and exclusion criteria

Articles met the following requirements were included: 1) patients suffered from tics treating with pharmacotherapies; 2) the comparison group could be placebo or another pharmacotherapies; 3) trials should be randomized controlled trials (RCTs), crossover and parallel group controlled trials. In addition, only the latest report would be included if the trial has been reported many times. Discontinuation research would be excluded.

### Data extraction and quality assessment

A pre-defined excel table was used to extract information about relevant characteristics of included studies such as participant, intervention, comparison, and outcome by two independent reviewers. Whether the trial was conducted in children or mixed participation (children and adult) was displayed as well. The primary efficacy outcome scale defined in this meta-analysis was the Yale Global Tic Severity Scale (YGTSS)[91], as it is the most frequently used scale with high acceptance, and the followings were defined as secondary outcomes: Clinical Global Impression Scale (CGI)[92], TS Global Scale (TSGS)[93], and TS Severity Scale (TSSS)[94]. In order to integrate the data extraction, some authors were contacted through emails for raw data. Anyone of the primary and secondary outcomes were extracted, if articles reported. Any disagreements were coordinated by a third reviewer to reach consensus. Risk of bias were evaluated based on methods recommended by Cochrane Handbook [95].

### Statistics

Overall estimates of pooled weighted mean difference (WMD) with 95% confidence interval (CI) were calculated for each outcome measure from baseline to endpoint other than standard mean difference (SMD), because WMD could reflect the original effect size change of each outcome measure and it's easier to understand clinically. Heterogeneities among trials were evaluated by Q and I^2^ statistic. If the results were P < 0.1 and I^2^ > 50%, the existence of heterogeneity was predicted and random effects model was used to summarize WMD and 95% CI (high heterogeneity defined as I^2^ >75%). If not, fixed effects model was used. In addition, sensitivity analysis would be performed by removing individual trial to test the reliability of findings. If any of the outcome included 10 or more studies, publication bias would be examined by funnel plot and Egger's test according to the recommendation of Cochrane Handbook [95, 96].

The above analyses were conducted with the help of STATA 11.0 (Stata Corporation, College Station, TX).

## CONCLUSIONS

In summary, there were effective pharmacological treatments for the management of patients with tics, especially atypical antipsychotic agents and alpha-2 adrenergic agonist agents. And alpha-2 adrenergic agonist agents were associated with the optimal weigh between efficacy and safety. However, the limited trials and sample sizes discounted these findings. Future better studies are necessary to ascertain them. In clinical practice, the choice of pharmacological treatments should balance overall advantage and disadvantage.

## SUPPLEMENTARY MATERIALS FIGURES AND TABLES




