# Supplementary material for: Pharmacotherapies to tics: a systematic review
Source: Oncotarget. 2018 Jun 15;9(46):28240–66. doi: 10.18632/oncotarget.25080 (PMC6021346; doi:10.18632/oncotarget.25080)
Supplement: Supplementary file 1 [file oncotarget-09-28240-s001.pdf]

Pharmacotherapies to tics: A systematic review

SUPPLEMENTARY MATERIALS

Supplementary appendix 1: Adverse effects of included pharmacological interventions

Supplementary Table 1: Adverse effects of Alpha-2 adrenergic agonist agent

Systems	Guanfacine	Clonidine
**Neurological and psychiatric symptoms**	Fatigue/sleepiness:25%(3/12);<br>Headache and Fatigue:8.33%(1/12);<br>Bad dreams:8.33%(1/12);	Fatigue:75%(3/4);<br>Sedation:41.67%(5/12)-57%;<br>Irritability:33%-75%(3/4);<br>Restlessness:27%;<br>Worsening of tics:26.47%(9/34);<br>Sad/depressed:25%(1/4);<br>Sleepwalking:25%(1/4);<br>Insomnia:0.31%(1/326)-50%(2/4);<br>Somnolence:0.31%(1/326)- 50%(2/4);<br>Dizziness:0.31%(1/326)-43%;
**Gastrointestinal tract**	Constipation:11.8%(2/17);<br>Loss of appetite:11.8%(2/17);	Stomachache:50%(2/4);
**cardiovascular tract**		Abnormal ECG:0.62%(2/326);
**Skins**		Rashes:0.92%(3/326);
**Others**	Dry mouth:23.5%(4/17);	Aggression:75%(3/4);<br>Stiffness:8.33%(1/12);<br>Dry mouth:8.33%(1/12)-57%;

ECG: Electrocardiograph.

Supplementary Table 2: Adverse effects of Anticonvulsant

Systems	Levetiracetam	Topiramate
**Neurological and psychiatric symptoms**	Irritability:66.67%(4/6);<br>Anxious:50%(3/6);<br>Sad/depressed:33.33%(2/6);<br>Hyperactive:33.33%(2/6);<br>Insomnia:33.33%(2/6);<br>Tired/sleepy:16.67%(1/6)-33.33%(2/6);<br>Fatigue:16.67%(1/6);<br>Dizzy:16.67%(1/6);	Headache:20%(3/15);<br>Drowsiness/hypersomnia:13%(2/15);<br>Cognitive slowing:7%(1/15);
**Gastrointestinal tract**	Loss of appetite:16.67%(1/6);	Diarrhoea:20%(3/15);<br>Abdominal pain:13%(2/15);
**Urogenital**		Kidney stone:7%(1/15);
**Others**	Aggression:33.33%(2/6);<br>Dry mouth:16.67%(1/6);	

Supplementary Table 3: Adverse effects of Antidepressant

Systems	Fluoxetine	Fluvoxamine	Desipramine	Deprenyl
**Neurological and psychiatric symptoms**	Motor restlessness:58.3%(7/12); Insomnia:41.7%(5/12); Fatigue:16.7%(2/12)-40%(2/5); Increased motor tics:16.7%(2/12); Dizziness:8.3%(1/12); Tremor:8.3%(1/12); Anxiety:8.3%(1/12);	Apathy:18.18%(2/11); Akathisia:18.18%(2/11); Hypomania:9.09%(1/11); Dystonia:9.09%(1/11);	Dysphoria/mood changes:24%(5/21); Difficulty sleeping:19%(4/21); Headache:10%(2/21); Unsteadiness/ dizziness:5%(1/21); Sedation:5%(1/21); Motion sickness:5%(1/21);	Drowsiness:38.46%(5/13); Agitation:30.77%(4/13); Headache:30.77%(4/13); Irritability:15.38%(2/13);
**Gastrointestinal tract**	Decreased appetite:25.0%(3/12); Diarrhea:25.0%(3/12);	Mild transient nausea:36.36%(4/11);	Increased thirst/dry mouth:10%(2/21); Nausea:5%(1/21); Constipation:5%(1/21); Heart burn:5%(1/21); Stomachache:5%(1/21); Indigestion:5%(1/21);	Nausea:30.77%(4/13); Diarrhea:7.69%(1/13);
**Eye areas**	Blurred vision:8.3%(1/12);		Blurred vision:5%(1/21);	
**cardiovascular tract**	Nose bleeds:16.7%(2/12);			
**Skins**			Rash:4.76%(1/21);	Rash:7.69%(1/13)

Supplementary Table 4: Adverse effects of Antipsychotic agent

See Supplementary File 1

Supplementary Table 5: Adverse effects of Cannabis

Systems	Delta 9-tetrahydrocannabinol
**Neurological and psychiatric symptoms**	Tiredness, dry month, dizziness, and muzziness:41.67%(5/12);

Supplementary Table 6: Adverse effects of CNS stimulant/plus Alpha-2 adrenergic agonist agent

systems	Methylphenidate	Dextroamphetamine	Methylphenidate plus Clonidine
**Neurological and psychiatric symptoms**	Worsening of tics:21.62%(8/37); Insomnia:10%(2/20);	Insomnia:50%%(10/20);	Worsening of tics:18.18%(6/33);
**Gastrointestinal tract**	Appetite suppression with transient weight loss:15%(3/20);	Appetite suppression with transient weight loss:20%(4/20);	
**Others**	Transient obsessive-compulsive symptoms:25%(5/20);	Transient obsessive-compulsive symptoms:5%(1/20);	

Supplementary Table 7: Adverse effects of Dopaminergic agent

Systems	Pergolide	Pramipexole	Talipexole
**Neurological and psychiatric symptoms**	Headaches:43%; Insomnia:16%; Syncope:8.33%(1/12); Sedation:8.33%(1/12); Dizziness:8.33%(1/12); Irritability:8.33%(1/12) ;	Headache:27.9%(12/43); Fatigue:9.3%(4/43);	Fatigue:75%(6/8); Dizziness:25%(2/8); Insomnia: 12.5% (1/8); Headache:12.5%(1/8); Syncope: 12.5% (1/8);
**Gastrointestinal tract**	Gastrointestinal adverse events :68%; Nausea:8.33%(1/12); Stomachache:8.33%(1/12);	Nausea:18.6%(8/43); Vomiting:11.6%(5/43);	Nausea:8.33%(1/8);
**Skins**	Rashes:11%;		
**Others**		Myalgia:9.3%(4/43);	Cold sweats:12.5% (1/8);

Supplementary Table 8: Adverse effects of Gamma-aminobutyric acid-B receptor agonist

Systems	Baclofen
**Neurological and psychiatric symptoms**	Anxiety:11.11%(1/9);<br>Headache:11.11%(1/9);
**Gastrointestinal tract**	Stomach pains or nausea:22.22%(1/9);<br>Constipation:11.11%(1/9);

Supplementary Table 9: Adverse effects of Glutamate agonist

Systems	D-serine	N-Acetylcysteine
**Neurological and psychiatric symptoms**	Irritability, moodiness:33%(2/9); Fatigue:22%(2/9); Lightheadedness, dizziness:11%(1/9); Sleeplessness:11%(1/9);	Headache:6%(1/17);
**Gastrointestinal tract**	Gastrointestinal upset:33%(3/9); Diarrhea:11%(1/9);	

Supplementary Table 10: Adverse effects of Glutamate antagonist

Systems	Riluzole
**Neurological and psychiatric symptoms**	Fatigue:20%(2/10);<br>Lightheadedness, dizzines:20%(2/10);<br>Headache:20%(2/10);<br>Irritability, moodiness:10%(1/10);<br>Sleeplessness:10%(1/10);
**Gastrointestinal tract**	Gastrointestinal upset:20% (2/10);<br>Diarrhea:20%(2/10);

Supplementary Table 11: Adverse effects of Selective norepinephrine reuptake inhibitor

Systems	Atomoxetine
**Neurological and psychiatric symptoms**	Headache:21.1%(16/76)-21.3%(13/61);<br>Fatigue:11.8%(9/76);
**Gastrointestinal tract**	Nausea:18%(11/61)-15.8%(12/76);<br>Decreased appetite:15.8%(12/76)- 18%(11/61);<br>Vomiting:15.8%(12/76)-16.4%(10/61);<br>Upper abdominal pain:9.2%(7/76)-11.5%(7/61);<br>Diarrhea:3.9%(3/76);
**Respiratory tract**	Cough:4.9%(3/61)-5.3%(4/76);<br>Pharyngitis:3.9%(3/76)-4.9%(3/61);

Supplementary Table 12: Adverse effects of Smoking cessation agent

Systems	Nicotine patch
**Neurological and psychiatric symptoms**	Headache:49%(17/35);<br>Dizziness:23%(8/35)-26%(9/35);<br>Lethargy:17%(6/35)-23%(8/35);<br>Anger outbursts:17%(6/35);<br>Sweating:11%(4/35);<br>Akathisia:11%(4/35);<br>Difficulty arousing from sleep:9%(3/35);<br>Irritability:9%(3/35);<br>Difficulty staying asleep:6%(2/35);
**Gastrointestinal tract**	Nausea:71%(25/35);<br>Vomiting:40%(14/35);<br>Abdominal pain:20%(7/35);
**Skins**	Itching:57%(20/35);

Supplementary Table 13: Adverse effects of Traditional Chinese medicine/plus Antipsychotic agent

Systems	Ningdong Granule	5-Ling Granule	Ningdong Granule plus Haloperidol
**Neurological and psychiatric symptoms**		Dizziness:0.8%(3/362); Sleep disturbance:0.8%(3/362); Physical tiredness:0.6%(2/362); Headache:0.3%(1/362);	Drowsiness:5%(3/60); Lassitude:3.33%(2/60);
**Gastrointestinal tract**	Poor appetite:6.06%(2/33); Constipation:3.03%(1/33);	Diarrhea:2.5%(9/362); Reduced appetite:1.7%(6/362); Nausea/ vomiting:1.1%(4/362);	Poor appetite:5%(3/60);
**Respiratory tract**		Upper respiratory tract infection:11%(40/362);	
**Others**		Lab testing abnormality:1.9%(7/362);	

Supplementary Table 14: Adverse effects of 5HT3-receptor antagonists

Systems	Ondansetron	Metoclopramide
**Neurological and psychiatric symptoms**		Sedation:21.43%(3/14);<br>Dysphoria/mood changes:7.14%(1/14);
**Gastrointestinal tract**	Abdominal pain:6.67%(1/15);<br>Gastrointestinal complaints:6.67%(1/15);	Increased appetite:21.43%(3/14);
