# Supplementary material for: Pharmacotherapies to tics: a systematic review
Source: Oncotarget. 2018 Jun 15;9(46):28240–66. doi: 10.18632/oncotarget.25080 (PMC6021346; doi:10.18632/oncotarget.25080)
Supplement: Supplementary file 2 [file oncotarget-09-28240-s002.docx]

**Table 4 Adverse effects of Antipsychotic agent**

**Typical Neuroleptics**

| **Systems** | **Haloperidol** | **Pimozide** | **Sulpiride** |
| --- | --- | --- | --- |
| **Neurological and psychiatric symptoms** | Akinesia:50%(9/18);  Drowsiness:13.33%(4/30);  Lassitude:10%(3/30)-11.11%(2/18);  Akathisia:5.56%(1/18)-36.67%(2/30);  Central nervous system acute dystonia:5.56%(1/18);  Cognitive dulling:5.56%(1/18); | Somnolence:41.67%(10/24);  Fatigue:37.5%(9/24);  Akinesia:25%(5/20)-90%(18/20);  Postural rigidity:20%(4/20);  Akathisia:10%(2/20)-40%(8/20);  Insomnia:10%(2/20)-29.17%(7/24);  Depression:10%(2/20)-25%(6/24) ;  Slurred language:10%(2/20) ;  Phobia:10%(2/20);  Cognitive dulling:5%(1/20)-10%(2/20) ;  Irritability:5%(1/20)-10%(2/20) ;  Headache:5%(1/20)-8.33%(2/24);  Tremor:5%(1/20); | Depression:27.27%(3/11); |
| **Gastrointestinal tract** | Gastrointestinal tract Increased appetite:22.22%(4/18) ;  Weight gain:11.11%(2/18);  Poor appetite:3.33%(1/30);  Constipation:3.33%(1/30); | Constipation:20%(4/20);  Increased appetite:5%(1/20);  Diarrhea:5%(1/20);  Weight gain:5%(1/20); |  |
| **Eye areas** |  | Mydriasis:5%(1/20); |  |
| **Urogenital** |  | Impotence:15%(3/20);  Urogenital Difficulty urinating:5%(1/20);  Urinary frequency:5%(1/20); |  |
| **cardiovascular tract** |  | Abnormal ECG:10%(1/10); |  |
| **Musculoskeletal** |  | Injuries:25%(6/24);  Hyperkinesia:20.83%(5/24);  Neck spasm:5%(1/20);  Back tightness:5%(1/20);  Leg tightness:5%(1/20);  Facial tightness:5%(1/20); |  |
| **Others** |  | Expressionless facies:10%(2/20);  Handwriting change:5%(1/20);  Slouched posture:5%(1/20);  Gynecomastia:5%(1/20);  Xerostomia:5%(1/20); |  |

ECG:Electrocardiograph

**Atypical Neuroleptics**

| **Systems** | **Ziprasidone** | **Risperidone** | **Aripiprazole** | **Tiapride** | **Olanzapine** |
| --- | --- | --- | --- | --- | --- |
| **Neurological and psychiatric symptoms** | Somnolence:6.25%(1/16)  Akathisia:6.25%(1/16) | Depression:26.1%(6/23)-30.77%(8/26);  Tremor:26.1%(6/23);  Headache:17.4%(4/23)-19.23%(5/26);  Agitation:17.4%(4/23);  Somnolence:17.2%(5/29)- 46.15%(12/26);  Foggy thinking:12.5%(2/16);  Sedation:11.11%(1/9)-19%(3/16);  Insomnia:3.85%(1/26)-21.7%(5/23);  Fatigue:3.4% (1/29)-56.5%(13/23);  Dizziness:3.4% (1/29)-39.1%(9/23); | Headache:15.63%(5/32);  Drowsiness:12.5%(4/32)-25.8%(8/31);  Sedation:12.5%(4/32);  Fatigue:9.7%(3/31) ;  Extrapyramidal disorder:9.38%(3/32);  Akathisia:6.3%(2/32)-6.45%(2/31);  Insomnia:3.23%(1/31);  Irritability:3.23%(1/31);  Dizziness:3.13%(1/32)-6.45%(2/31); | Sleep disturbance:8.9%(11/123);  Physical tiredness:7.3%(9/123);  Dizziness:4.1%(5/123);  Headache:1.6%(2/123); | Drowsiness:25%(1/4) |
| **Gastrointestinal tract** |  | Increased appetite:27.6%(8/29)-44%(7/16);  Saliva increased:26.1%(6/23);  Weight increase:21.7%(5/23);  Abdominal pain:6.9%(2/29);  Decreased appetite:6.5%(1/16);  Nausea/vomiting:3.4%(1/29)-6.5% (1/16); | Decreased Appetite:12.9%(4/31);  Abdominal pain:9.7%(3/31);  Nausea:9.68%(3/31)-18.8%(6/32);  Increased appetite:6.3%(2/32)-25.8%(8/31);  Anorexia:6.3%(2/32);  Dyspepsia:3.1%(1/32); | Reduced appetite:1.6%(2/123);  Nausea/vomiting:1.6%(2/123); |  |
| **Eye areas** |  | Blurred vision:10.3%(3/29)-12.5%(2/16);  Vision abnormal:4.4%(1/23); | Blurred vision:9.7%(3/31); |  |  |
| **Urogenital** |  | Diurnal Urinary incontinency:13.8%(4/29); |  |  |  |
| **cardiovascular tract** |  |  | Electrocardiogram QT prolonged:6.3%(2/32); |  |  |
| **Respiratory tract** |  | Influenza-like symptoms:8.7%(2/23); | Nasopharyngitis:12.5%(4/32);  Upper respiratory tract infection:3.1%(1/32); | Upper respiratory tract infection:8.1%(10/123); |  |
| **Skins** |  | Itches:10.3%(3/29) | Itches:3.2%(1/31) |  |  |
| **Others** |  | Stiffness:22.22%(2/9);  Mouth dry:21.7%(5/23);  Hypertonia:17.4%(4/23);  Libido increased:17.4%(4/23);  Social phobia:13%(2/16);  Erectile difficulties:13%(2/16);  Infection viral:8.7%(2/23)  Hyperkinesia:7.69%(2/26)  Back pain:4.4%(1/23);  Injuries:3.85%(1/26); |  | Sweating:1.6%(2/123);  Lab testing abnormality:1.6%(2/123); |  |
